# Isolation and Characterization T4- and T7-Like Phages that Infect the Bacterial Plant Pathogen *Agrobacterium tumefaciens*

**DOI:** 10.3390/v11060528

**Published:** 2019-06-07

**Authors:** Hedieh Attai, Pamela J.B. Brown

**Affiliations:** Division of Biological Sciences, University of Missouri, Columbia, MO 65211, USA; hattai@ucsd.edu

**Keywords:** *Agrobacterium tumefaciens*, bacteriophage, phage, biocontrol

## Abstract

In the rhizosphere, bacteria–phage interactions are likely to have important impacts on the ecology of microbial communities and microbe–plant interactions. To better understand the dynamics of Agrobacteria–phage interactions, we have isolated diverse bacteriophages which infect the bacterial plant pathogen, *Agrobacterium tumefaciens*. Here, we complete the genomic characterization of *Agrobacterium* *tumefaciens* phages Atu_ph04 and Atu_ph08. Atu_ph04—a T4-like phage belonging to the *Myoviridae* family—was isolated from waste water and has a 143,349 bp genome that encodes 223 predicted open reading frames (ORFs). Based on phylogenetic analysis and whole-genome alignments, Atu_ph04 is a member of a newly described T4 superfamily that contains other *Rhizobiales*-infecting phages. Atu_ph08, a member of the *Podoviridae* T7-like family, was isolated from waste water, has a 59,034 bp genome, and encodes 75 ORFs. Based on phylogenetic analysis and whole-genome alignments, Atu_ph08 may form a new T7 superfamily which includes *Sinorhizobium* phage PCB5 and *Ochrobactrum* phage POI1126. Atu_ph08 is predicted to have lysogenic activity, as we found evidence of an integrase and several transcriptional repressors with similarity to proteins in transducing phage P22. Together, this data suggests that *Agrobacterium* phages are diverse in morphology, genomic content, and lifestyle.

## 1. Introduction

*Agrobacterium tumefaciens* is a plant pathogen that causes damage to crops worldwide [1]. This gram-negative bacterium transforms plant cells, which results in overproliferation of host cells, causing crown gall disease in the form of tumors that block the plant from receiving proper nutrients. The interactions between *Agrobacterium* and plants have been studied extensively, leading to innovations in plant biotechnology [2,3]. In contrast, little is known about the natural predators of *Agrobacterium*. Studies of bacteriophages that prey upon bacterial plant pathogens such as *Agrobacterium* should reveal effective biocontrol strategies for host cell killing that can be exploited to limit phytopathogenesis [4,5]. With the rise of antibiotic resistant bacteria, there has been an increased interest in phage research; however, the diversity of phages that infect soil bacteria is undersampled relative to phages of human pathogens and marine environments [6,7]. Understanding the diversity of phages in soil is important because of their impact on host populations, community interactions, and biogeochemical cycles [8].

Here, we sought to further explore the diversity of phages that infect *Agrobacterium tumefaciens.* Currently, there are four characterized lytic phages that infect *Agrobacterium*: 7-7-1 [9], Atu_ph02 and Atu_ph03 [10], and Atu_ph07—a jumbo phage [11]. Phages 7-7-1 and Atu_ph07 are T4-like *Myoviridae* and Atu_ph02 and Atu_ph03 are T7-like *Podoviridae*. Here, we report characteristics of 2 additional phages, Atu_ph04 and Atu_ph08, and compare them to related phages, including the extensively characterized *Escherichia* phages T4 [12,13] and P1.

## 2. Materials and Methods

### 2.1. Bacterial Strains and Culture Conditions

Strains used in this study are shown in Table 1. *Agrobacterium tumefaciens* strains were cultured in Lysogeny Broth (LB), with the exception of *A. tumefaciens* strain LBA4404, which was grown in yeast mannitol (YM) medium. *Agrobacterium vitis* was cultured using potato dextrose media (Difco), *Rhizobium rhizogenes* was grown in mannitol glutamate yeast (MGY) medium, and *Caulobacter crescentus* was grown in peptone-yeast extract (PYE) medium [14]. These strains were grown at 28 °C. *Escherichia coli* was grown in LB at 37 °C. Liquid cultures were grown with shaking and solid medium was prepared with 1.5% agar.

### 2.2. Phage Isolation and Purification

Phage Atu_ph04 was isolated from an effluent sample from a waste water treatment plant in Columbia, MO, while Atu_ph08 was isolated from a waste water sample from Reno, Nevada. *A. tumefaciens* strain C58 was used as a host strain, using the multiple-enrichment isolation method as described previously [10,20].

### 2.3. Plaque Assays

Whole-plate plaque assays were performed with the soft agar overlay method [10]. Briefly, 100 µL cells, grown at an optical density of 600 nm (OD_600_) of ~0.2 and diluted to OD_600_ of 0.05, were mixed with 100 µL phage for 15 min at room temperature prior to dilution to allow attachment. This mixture of cells and phage were serially diluted in LB and added to 3 mL of melted 0.3% LB-soft agar. The solution was then overlaid onto a 1% LB agar plate and swirled for even distribution. For host range testing, serial dilutions of phage were spotted onto a bacterial lawn. A mixture of 100 µL cells (OD_600_ of ~0.2) and 0.3% LB-soft agar was overlaid onto a 1% LB agar plate. Once the cells solidified, 5 µL of phage dilutions were spotted onto the soft agar. Plates were incubated for 1–2 days to allow plaque formation.

### 2.4. Preparation of Virion DNA, Genome Sequencing, and Genome Assembly

DNA was isolated from purified virions using phenol–chloroform extraction as described previously [10]. Libraries for genome sequencing were constructed from virion DNA following the manufacturer’s protocol and reagents supplied in Illumina’s TruSeq DNA PCR-free sample preparation kit (FC-121-3001) [10]. The purified library was quantified using a KAPA library quantification kit (KK4824), and library fragment sizes were confirmed by Fragment Analyzer (Agilent, Santa Clara, CA, USA). Libraries were diluted, pooled, and sequenced using a paired-end 75-base read length according to Illumina’s standard sequencing protocol for the MiSeq. Library preparation and sequencing were conducted by the University of Missouri DNA core facility.

### 2.5. DNA Restriction Analysis

Phage genomic DNA was digested with restriction endonucleases from New England Biolabs using the standard protocol. All reactions contained 500 ng DNA, which was incubated for 2 h at 37 °C. Digested DNA was analyzed on a 0.7% agarose gel. Gel electrophoresis was performed at 100 V for 1 h and stained with SYBR Safe DNA Gel Stain (Thermo Scientific, Waltham, MA, USA).

### 2.6. Growth Curves

Growth curves were performed by growing bacteria at a starting OD_600_ of 0.05 in LB. Cells were mixed with purified phage in liquid medium at the MOIs indicated. Cell growth was measured by the culture turbidity, represented by the absorbance at OD_600_. Measurements were taken every 10 min for 36 h. Cells were grown at 28 °C and shaken for 1 min prior to each reading. The OD_600_ was measured using a BioTek Synergy H1 Hybrid reader. Results were taken in quadruplicate and averaged.

### 2.7. Transmission Electron Microscopy

Virion morphology was observed by applying a small volume of concentrated purified virions onto a freshly glow-discharged, carbon-coated TEM grid and negatively stained with 2% Nano-W (Nanoprobes, LLC, Brookhaven, NY, USA) or 2% uranyl acetate. Specimens were observed on a JEOL JEM-1400 transmission electron microscope at 120 kV. Capsid diameters of Atu_ph04 (*n* = 103 virions) and Atu_ph08 (*n* = 61 virions), as well as tails of Atu_ph04 (*n* = 15 virions) and Atu_ph08 (*n* = 15 virions) were measured using ImageJ (v.2.0.0) [21].

### 2.8. Genome Annotation

The sequences were annotated by the RAST server [22] and ORFs with no homology in the database, or ORFans, were defined as having an e-value greater than 1 × 10^−3^ by PSI-BLAST v 2.8.1 [23]. All gene products were analyzed by TMHMM [24]. The presence of tRNAs was detected by tRNAscan-SE (version 2.0) [25]. G + C content was analyzed by Geneious (v.11.0.5) [26]. Pairwise (%) nucleotide identity was determined using the Mauve plugin in Geneious [27].

### 2.9. Phylogenetic Analysis

Homologs of the large terminase subunit in Atu_ph08 and portal vertex protein in Atu_ph04 were identified by BLASTp using an *E*-value cutoff of 1 × 10^−3^. Protein alignment was performed by Geneious using ClustalW (v.2.1) and the BLOSUM matrix [26,28]. Maximum-likelihood trees based on phylogeny (PhyML) were built using a Geneious plugin with 100 bootstrap models [29].

### 2.10. GenBank Accession Number

The genome sequences of *Agrobacterium* phages Atu_ph04 and Atu_ph08 are available in GenBank under accession numbers MF403007 and MF403009, respectively.

## 3. Results and Discussion

### 3.1. Phage Atu_ph08 has Higher Lytic Activity than Atu_ph04

**Figure 1:**
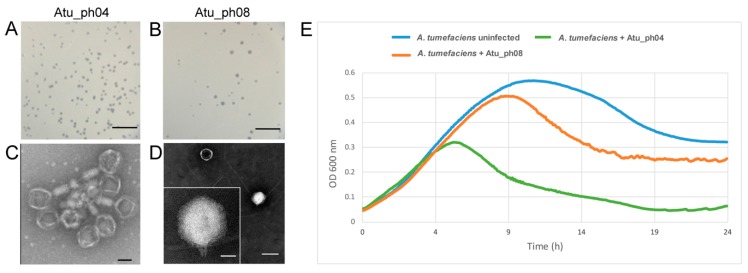
Characterization of Atu_ph04 and Atu_ph08. Plaque assays of Atu_ph04 (**A**) and Atu_ph08 (**B**). Scale bars represent 10 mm. Transmission electron microscopy of (**C**) Atu_ph04 shows it is in the *Myoviridae* family. Scale bar represents 100 nm. (**D**) Atu_ph08 is in the family *Podoviridae*. Scale bar (right) represents 100 nm and scale bar in inset represents 25 nm. (**E**) Growth curve of *A. tumefaciens* C58 cells growing in the presence and absence of phage at an MOI of 0.001.

Waste water includes agricultural runoff, and provides an enriched mixture of bacterial populations, making this a prime environment for isolation of bacteriophages. We isolated phages that infect *A. tumefaciens* from waste water using a phage enrichment protocol as described previously [10]. Infection of *A. tumefaciens* C58 with Atu_ph04 or Atu_ph08 results in the formation of small, clear plaques (Figure 1A) or larger, clear plaques (Figure 1B), respectively. Negative-staining transmission electron microscopy (TEM) of Atu_ph04 reveals an icosahedral head and tail (Figure 1C), classifying Atu_ph04 in the family *Myoviridae* [30]. The average capsid head diameter of Atu_ph04 is 84.7 nm and its tail length is 79.8 nm. TEM of Atu_ph08 reveals the presence of an icosahedral head with an average diameter of 65.0 nm and a short, stubby tail with a length of 21.9 nm (Figure 1D), indicating that this phage belongs to the *Podoviridae*.

Growth curves of *A. tumefaciens* strain C58 infected with Atu_ph04 and Atu_ph08 at an MOI of 0.001 reveals that Atu_ph04 begins to exhibit lethal activity at 4 h post-infection, whereas the modest lytic activity of Atu_ph08 is observable after 8 h post-infection (Figure 1E). While both phages exhibit lytic activity, Atu_ph04 would be preferred for biocontrol purposes because it significantly reduces cell turbidity.

### 3.2. Host Ranges of Atu_ph04 and Atu_ph08 are Limited to A. tumefaciens Strains

Host range was determined by performing plaque assays of phage dilutions and is summarized in Table 2. Atu_ph04 causes lysis of most C58-derived *A. tumefaciens* strains, including C58, EHA101, EHA105, and GV3101, but does not infect AGL-1. Furthermore, Atu_ph04 is able to lyse NTL4 and LBA4404 but unable to infect *A. tumefaciens* Chry5 or other bacterial species. Atu_ph08 lyses C58-derived *A. tumefaciens*, however it is only moderately infective in AGL-1. Atu_ph08 does not infect Chry5 or other bacterial species. This host range is comparable to the range of other *A. tumefaciens*-infecting phages described. The narrow range suggests that Atu_ph04 and Atu_ph08 will not disrupt other, beneficial bacterial strains in the rhizosphere, an important consideration when selecting phages for biocontrol.

### 3.3. Genomic Characteristics of Atu_ph04

**Figure 2:**
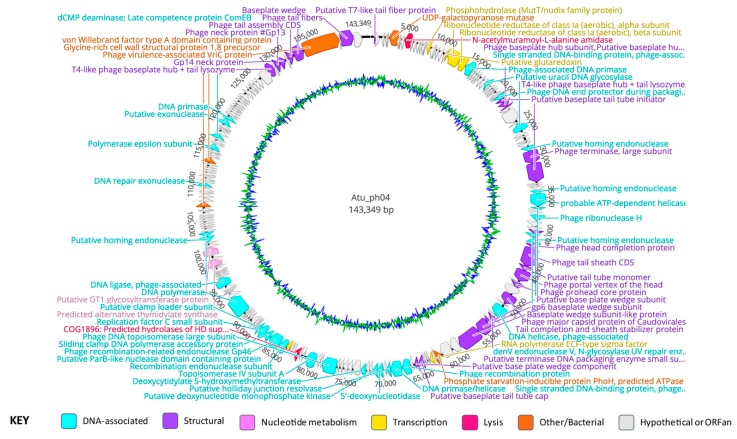
Genome annotation of Atu_ph04, color-coded by functional annotation. G + C content represented by inner circle: AT = green; GC = blue.

The genome of Atu_ph04 is 143,349 bp in length, with a G + C content of 49.4% (Figure 2, Supplementary Table S1, Table 3). Interestingly, attempts to digest the Atu_ph04 genomic DNA with nine different restriction enzymes failed, despite the presence of the restriction sites in the genome sequence, suggesting that the DNA may be modified (Supplementary Figure S1). The genome of Atu_ph04 encodes 223 open reading frames (ORFs), of which, 73 have predicted functions; 83 are ORFans, meaning they have no obvious homologs; and 67 conserved hypothetical proteins. Atu_ph04 only encodes one predicted tRNA, but its anticodon is undetermined, as predicted by tRNAscan-SE v 2.0 [25].

Of the 73 gene products with predicted functions encoded by Atu_ph04, many include structural proteins such as the portal vertex of the head (gp72), the major capsid protein (gp76), and a T4-like phage large terminase (gp53). The Atu_ph04 major capsid protein shares 76% identity with *Sinorhizobium* phage phiM9 major head subunit, gp23, as characterized by Johnson et al. [31]. Atu_ph04 also encodes DNA synthesis proteins, including DNA topoisomerase (gp110 and gp113), nucleotide metabolism proteins, such as ribonucleotide reductase of class 1a alpha (gp24) and beta subunits (gp25), and proteins involved in translation, like RNA polymerase sigma factor (gp89 and 119).

### 3.4. Phylogenetic Analysis Shows Atu_ph04 is Closely Related to T4-Like Sinorhizobium Phage phiM9 and Rhizobium Phage vB_RleM_P10VF

**Figure 3:**
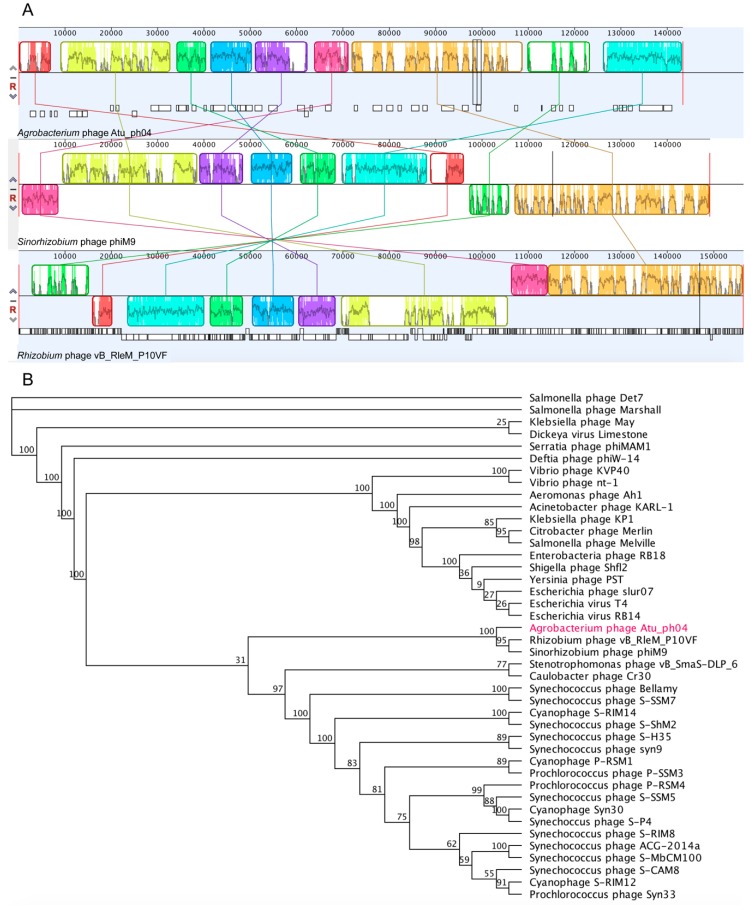
Phylogenetic analysis of Atu_ph04 with its relatives. (**A**) Mauve genome alignment of Atu_ph04, *Sinorhizobium* phage phiM9, and *Rhizobium* phage RleM_P10VF. (**B**) Phylogenetic tree of portal vertex protein.

Phage Atu_ph04 shares pairwise identity with *Rhizobium* phage vB_RleM_P10VF (21.6%) and *Sinorhizobium* phage phiM9 (19.7%), and whole-genome alignments constructed using Mauve [27] reveal that the three genomes contain blocks of genomic synteny (Figure 3A), suggesting that Atu_ph04 joins this recently-described group of T4 superfamily phages [31]. This analysis is consistent with the phylogenetic tree built using an alignment of the portal vertex protein (Figure 3B). This group of rhizophages is clustered into a larger group of cyanophages and *Synechococcus* phages. Comparative analysis of the gene products of Atu_ph04 with those of several representative T4-like phages confirms a relatively high degree of gene conservation among *Rhizobium* phage vB_RleM_P10VF and *Sinorhizobium* phage phiM9 (Supplementary Table S2).

### 3.5. Atu_ph04 is a T4-like Phage but Lacks Several T4 Core Proteins

Though Atu_ph04 is placed in the T4 superfamily, Atu_ph04 only shares 4.5% pairwise identity with Enterobacteria phage T4. To determine the relationship between Atu_ph04 and T4, we performed a comparative analysis matching T4 core proteins with the Atu_ph04 genome (Supplementary Table S3). The genome of Atu_ph04 encodes putative homologs of 14 of the 22 T4 core proteins (with an *E*-value > 1 × 10^−3^); however, it is missing key T4 core proteins, including some structural proteins. Though the Atu_ph04 genome encodes a T4-like gp21, the prohead core protein, it does not encode gp22, another prohead core protein that is essential in phage T4 [12]. Similar to phages phiM9 and vB_RleM_P10VF, Atu_ph04 also has a split T4 gp5 baseplate hub protein (gp54 and 213). The Atu_ph04 genome also lacks obvious homologs of T4-like tail fibers (T4 gp34 and 36). The absence of T4-like tail fibers in the Atu_ph04 genome (Supplementary Table S3) may be compensated by the presence of gp222, a predicted tail fiber protein and that is conserved in phiM9 and vB_RleM_P10VF (Supplementary Table S2). This difference in tail fiber proteins likely allows this group of rhizophages to infect a different host than T4 does.

Another feature of Atu_ph04, phiM9, and vB_RleM_P10VF genomes is the lack of genes encoding T4 protein gp33, which is involved in late transcription. Instead, it is hypothesized that phiM9 and vB_RleM_P10VF encode an RpoE stress response sigma factor, which compensates for the missing protein [31]. In the Atu_ph04 genome, not only is T4 protein gp33 missing, but the core sigma factor for late transcription protein gp55 is also not encoded. The Atu_ph04 genome encodes a DNA-directed RNA polymerase RpoE sigma factor (gp89) that shares 20.3% pairwise identity with the sigma factor in phiM9. It also encodes gp119, a putative sigma factor for late transcription, which shares 49% identity with the one encoded by phiM9. Additionally, the Atu_ph04 genome encodes T4 core protein NrdA (gp24), the alpha subunit of ribonucleotide reductase class 1a, which is involved in nucleotide metabolism. Yet, instead of *nrdB*, which encodes the beta subunit in T4, it encodes a presumably diverged class 1a ribonucleotide reductase—beta subunit homolog (gp25)—adjacent to its alpha partner. Together, these data suggest that the rhizophages have diverged from the T4-phages with respect to regulation of transcription throughout the phage replication cycle and nucleotide metabolism.

### 3.6. Major Gene Categories of Atu_ph04

The Atu_ph04 genome encodes 25 predicted structural gene products, including two putative tail fiber proteins (gp1 and 222), four tail completion and sheath proteins (gp66, 70, 71, and 218), 11 baseplate subunits (gp41, 42, 43, 54, 82, 83, 84, 93, 94, 213, and 219), four capsid head proteins (gp69, 72, 74, and 76), two terminase proteins (gp53 and 80), and two neck proteins (gp215 and 216). Protein VrlC (gp220) is predicted to be responsible for the structure of double-layered, or double ring-like, baseplates [32,33], which are a feature of some T4-like phages but not T4 itself.

Atu_ph04 has an abundance of genes involved in DNA replication, repair, and recombination. It encodes 34 DNA-associated proteins involved in DNA replication, repair, and recombination. The DNA replication proteins include two DNA primases (gp26 and 195), single-stranded DNA binding proteins (gp47 and 67), ribonuclease H (gp63) [34], DNA helicase (gp78), two topoisomerase subunits (gp110 and 113), and three sliding clamp loader subunits (gp122, 123, and 124). The DNA polymerase is predicted to be gp133. There is a cluster of DNA-associated proteins: DNA primase/helicase (gp97), a putative holliday junction resolvase (gp98), 5’-deoxynucleotidase (gp100); a deoxynucleotide monophosphate kinase (gp101); and deoxycytidylate 5-hydroxymethyltransferase (gp104).

The presence of three putative homing endonucleases (gp52, 58, and 68) in close proximity to the large terminase (gp53) is consistent with the hypothesis that these endonucleases are involved in DNA packaging [35]. Gp60 shares similarity with T4 protein DenV, which is responsible for the removal of pyrimidine dimers caused by UV damage, a process necessary for DNA repair [36].

Several proteins involved in nucleotide metabolism are often encoded by phages. The Atu_ph04 genome encodes six proteins involved in this process. These include the MutT/Nudix family protein (gp17), a putative glutaredoxin (gp23), ribonucleotide reductase alpha (gp24) and beta (gp25) subunits, thymidylate synthase (gp145), and GT1 glycosyltransferase (gp148).

Atu_ph04 also encodes several genes that enhance the survival of their bacterial hosts. One such example is the phosphate starvation-inducible protein PhoH (gp87), which is suggested to enhance the phosphate metabolism in the host under stress [37]. Another bacterial gene product (gp6) encodes UDP-galactopyranose mutase, which is involved in the synthesis of the essential bacterial cell wall component, galactofuranose [38]. Finally, Atu_ph04 encodes two putative lysis proteins: gp10, which is an N-acetylmuramoyl-L-alanine amidase, and gp116, which is a predicted hydrolase of the conserved HD superfamily consistent with our classification of Atu_ph04 as a lytic phage.

### 3.7. Atu_ph08 Genomic Summary

**Figure 4:**
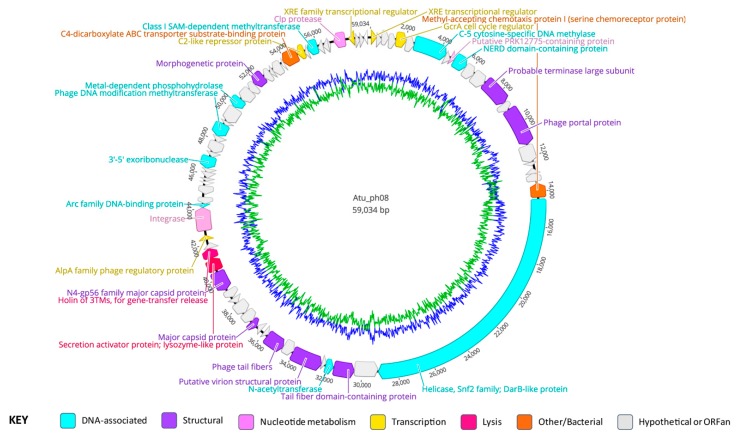
Genome annotation of Atu_ph08, color-coded by functional annotation. G + C content represented by inner circle: AT = green; GC = blue.

The genome of Atu_ph08 is 59,034 bp in length, with a G + C content of 59.7% (Figure 4, Table 3, Supplementary Table S4). The Atu_ph08 genome encodes 75 ORFs, only three of which are ORFans (gp45, 63, and 75). Of the 75 ORFs, 43 encode conserved hypothetical proteins and 32 have predicted functions. Atu_ph08 does not contain any obvious tRNA-encoding genes.

### 3.8. Gene Organization of Atu_ph08

The Atu_ph08 genome encodes eight predicted structural proteins (Figure 4, purple arrows), including two potential major capsid proteins (gp31 and 36), the tail fiber proteins (gp23 and 28), the portal protein (gp15), and the large terminase (gp13). Remarkably, the Atu_ph08 genome does not encode any gene products involved in DNA replication, such as DNA polymerase, with the exception of the DarB-like gp21, suggesting that it may use host machinery to replicate its DNA. The genome does encode several gene products predicted to be involved in DNA modification. These include gp7, which is a cytosine-specific DNA methylase and a NERD domain-containing protein (gp10), predicted to be involved in DNA processing [39]. Other DNA modification proteins include N-acetyltransferase (gp24), 3’-5’ exoribonuclease (gp49), methyltransferase (gp53), a metal-dependent phosphohydrolase (gp56), and a class I SAM-dependent methyltransferase (gp67).

Atu_ph08 also encodes transcription regulators, including the GcrA cell cycle regulator (gp5), which activates transcription at methylated promoter sequences by interacting with RNA polymerase, previously characterized in *Caulobacter crescentus* [40]. The putative GcrA regulator in the Atu_ph08 genome is 89.74% identical to a hypothetical protein (WP_080842116.1) in *Agrobacterium* genomospecies 3. The GcrA protein is conserved within the Alphaproteobacteria [41], as well as phiCbK-like *C. crescentus* phages [42], suggesting that phages may have acquired the gene encoding this protein from their hosts, potentially enabling the phages to upregulate host DNA replication machinery.

There are two predicted genes involved in posttranslational modifications. Gp71 is predicted to be a Clp protease, and gp9 contains a PRK12775 domain, which is predicted to be involved in amino acid transport and metabolism.

### 3.9. Atu_ph08 has Some Features of a Temperate Phage and Shares High Homology with A. tumefaciens genomospecies 3

**Figure 5:**
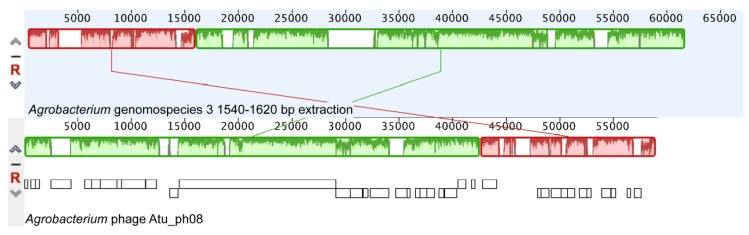
Mauve genome alignment of the 1540–1610 kbp region of *Agrobacterium* genomospecies 3 and Atu_ph08.

The genome of Atu_ph08 shares most of its genes with *A. tumefaciens* and *Rhizobium* species, leading us to hypothesize that Atu_ph08 and the Alphaproteobacteria have exchanged genes through horizontal gene transfer. Furthermore, the G + C content of the genomes of *A. tumefaciens* and phage Atu_ph08 are similar (~59%), in contrast with the G + C content of the other *Agrobacterium* phages, which are all lower. An initial analysis of the *Agrobacterium* genomospecies 3 strain CFBP 6623 genome (Accession number: NZ_LT009723) reveals the existence of three intact prophage regions and one incomplete prophage at the 1.5 million bp [43]. Mauve genome alignment of Atu_ph08 with this region in *Agrobacterium* genomospecies 3 strain CFBP 6623 (1,555,808–1,601,554 bp) revealed a 60.2% pairwise identity between the genomes (Figure 5).

Interestingly, while attempts to UV-induce lysogens from *A. tumefaciens* C58 cells infected with Atu_ph08 have been unsuccessful thus far, the Atu_ph08 genome encodes an integrase (gp41) and an XRE transcriptional regulator (gp1). The XRE transcriptional regulator belongs to a family of transcriptional regulators that contains Cro and cI repressors [44], suggesting that Atu_ph08 may exhibit lysogenic activity or be derived from an ancestor with lysogenic activity. The Atu_ph08 integrase shares 34% identity to the integrase encoded by *Salmonella* phage vB_SemP_Emek, which is a P22-like phage. P22 is a transducing phage that encodes the C2 repressor, so we sought to determine if the Atu_ph08 genome encodes a transcriptional repressor. Remarkably, gp65, annotated as a transcriptional regulator, shares 28% identity with the C2 repressor in vB_SemP_Emek. Directly upstream of the gene encoding the integrase is the gene encoding an Arc family phage regulatory protein (gp42), which acts as a transcriptional repressor in phage P22 [45]. Directly downstream of these genes is another peculiar gene encoding an AlpA family phage regulatory protein (gp40). AlpA has been characterized in *E. coli* to suppress sensitivity to UV light [46]. The presence of these genes strongly suggests that Atu_ph08 may be lysogenic and it should be explored as a candidate transducing phage for *A. tumefaciens*.

### 3.10. The Atu_ph08 Genome is Highly Syntenic with the Genome of the T7-Like Sinorhizobium Phage PBC5

**Figure 6:**
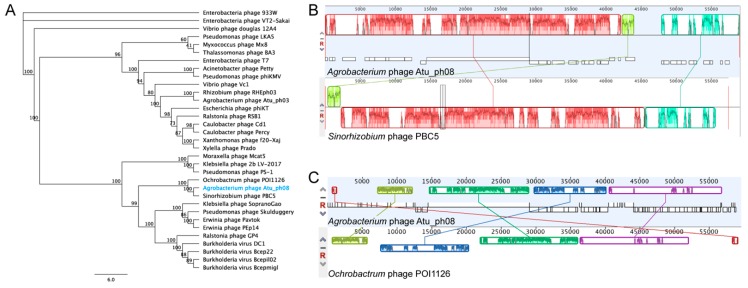
Relatives of Atu_ph08. (**A**) Phylogenetic tree of large terminase protein. Mauve genome alignment of Atu_ph08 with (**B**) *Sinorhizobium* phage PBC5 and (**C**) *Ochrobactrum* phage POI1126.

Phylogenetic analysis of Atu_ph08 reveals that it is closely related to *Sinorhizobium* phage PBC5 and *Ochrobactrum* phage POI1126. The Atu_ph08 genome shares 38.2% pairwise identity with *Sinorhizobium* phage PBC5 and 24.0% identity with *Ochrobactrum* phage POI1126. The large terminase tree (Figure 6A) shows that Atu_ph08 forms a distinct group with PBC5 and POI1126, and is placed within a larger group with T7-like *Burkholderia* phage Bcepmigl and *Erwinia* phage PEp14. These phages are distant relatives of the T7-superfamily of *Podoviridae* phages. Comparative analysis of the gene products of Atu_ph08 with those of several representative T7-superfamily phages confirms a high degree of gene conservation among *Sinorhizobium* phage PBC5 and *Ochrobactrum* phage POI1126 (Supplementary Table S5). The close relation to PBC5 and POI1126 are verified in the Mauve alignment of the genomes (Figure 6B). These alignments show evidence that genomic rearrangements have taken place among phages in this family. The mosaicism of phage genomes is a common result of horizontal gene transfer [47].

### 3.11. The Atu_ph08 Genome Encodes a DarB-like Protein, Commonly Found Among PBC5-Like Phages

The Atu_ph08 genome encodes a 4877 aa gene product (gp21), previously discussed in the context of this phage family in Gill et al. [48], which has four major domains that suggest it may have helicase and methylase activity (Supplementary Figure S2A). This unusually large gene product is described as a DarB homolog. DarB (defense against restriction) is an *Escherichia* phage P1 protein that protects the phage from host restriction enzymes *Eco*B and *Eco*K [49]. In phage P1, DarB is prepackaged inside the capsid, allowing DNA methylation to occur immediately upon infection, protecting the DNA from host killing by restriction [50,51].

The DarB-like protein in Atu_ph08 is 21.6% identical to the DarB-like protein of *Burkholderia* phage Bcep22 and is predicted to have both methyltransferase and helicase domains. Similar to Bcep22, Atu_ph08 does not have a DarA homolog encoded in the genome, which was thought to be required for DarB incorporation into the capsid. The DarB protein in Bcep22 contains a lytic transglycosylase domain in its N-terminal region. The Atu_ph08 DarB protein appears to have an N-terminal cell wall hydrolase domain followed by a peptidase domain.

This DarB-like protein appears to be conserved in several T7-like phages (Supplementary Figure S2B). A bioinformatic search for Atu_ph08 gene products conserved among *Agrobacterium* phages (Supplementary Table S6) found that *Agrobacterium* phages Atu_ph02 and Atu_ph03 also have a DarB-like protein. Since Atu_ph02 and Atu_ph03 share a host with Atu_ph08, acquisition of similar proteins to protect phage DNA from *A. tumefaciens* restriction and modification systems that destroy foreign DNA is plausible. Remarkably, DarB homologs are often found on mobile genetic elements, including the Ti plasmid of *A. tumefaciens*, suggesting that DarB likely confers a benefit to invading foreign DNAs [48].

### 3.12. The Atu_ph08 Genome Encodes a Putative Holin-Endolysin Cassette

The genome of Atu_ph08 encodes three possible gene products involved in cell lysis, which are consecutively located (gp37-9). The first, gp37, encodes a lysozyme-like domain. Directly adjacent, gp38 shares homology with a putative 3TM holin, named after a family of holins for gene transfer release with three transmembrane domains, encoded by Alphaproteobacterium *Mesorhizobium australicum*. All three genes are predicted to encode transmembrane domains—gp37 contains 1, gp38 contains 2, and gp39 contains 3. As holins are typically located in the inner membrane where they form a pore, it is likely that gp38 exhibits holin activity.

## 4. Conclusions

In this study, we characterize two additional *Agrobacterium* phages, which is important given the undersampling of phages from soil and rhizosphere environments. Despite sharing a common host, no conserved proteins were identified among all the *Agrobacterium* phage genomes, suggesting that the phages may not share mechanisms of host entry or lysis. Atu_ph04 forms a group with *Rhizobium* phage RleM_P10VF and *Sinorhizobium* phage phiM9, which are in the T4 superfamily, and Atu_ph08 is closely related to *Sinorhizobium* phage PBC5 and *Ochrobactrum* phage POI1126, which are T7-like. Through our comparative analysis, we found that Atu_ph08 may be a temperate phage, as it encodes several genes that are commonly expressed in phages that undergo the lysogenic cycle. Together, this data, along with previously published data on *Agrobacterium* phages, illustrates the diversity of phages that share a common host and provides examples of the breadth of genes these phages express, which can further our understanding of microbial diversity. Further studies are required to understand the impact these phages play in the environment where they reside.

## Figures and Tables

**Table 1:** Bacterial strains used in this study.

Strain or Plasmid	Relevant Characteristics	Growth Medium	Reference or Source
*A. tumefaciens* strains			
C58	Nopaline type strain; pTiC58; pAtC58	LB	[15]
EHA105	C58 derived, succinamopine strain, T-DNA deletion derivative of pTiBo542	LB	MU plant transformation core
EHA101	C58 derived, nopaline strain, T-DNA deletion derivative of pTiBo542	LB	MU plant transformation core
GV3101	C58 derived, nopaline strain	LB	MU plant transformation core
NTL4	C58 derived, nopaline-agrocinopine strain, ∆*tetRA*	LB	[16]
AGL-1	C58 derived, succinamopine strain, T-DNA deletion derivative of pTiBo542 ∆recA	LB	MU plant transformation core
LBA4404	Ach5 derived, octopine strain, T-DNA deletion derivative of pTiAch5	YM	MU plant transformation core
Chry5	Succinamopine strain, pTiChry5	LB	[17]
Other bacterial strains			
*A. vitis* S4	Vitopine strain, pTiS4, pSymA, pSymB	Potato dextrose	[18]
*Caulobacter crescentus* CB15	Alphaproteobacterium	PYE	[19]
*Escherichia coli* DH5α	Gammaproteobacterium	LB	Life Technologies

**Table 2:** Host range testing of Atu_ph04 and Atu_ph08.

Strain	Susceptibility to Phage ^1^	
	Atu_ph04	Atu_ph08
*A. tumefaciens* C58	S	S
*A. tumefaciens* EHA105	S	S
*A. tumefaciens* EHA101	S	S
*A. tumefaciens* GV3101	S	S
*A. tumefaciens* NTL4	S	S
*A. tumefaciens* AGL-1	R	I
*A. tumefaciens* LBA4404	I	I
*A. tumefaciens* Chry5	R	R
*A. vitis* S4	R	R
*C. crescentus* CB15	R	R
*E. coli* DH5α	R	R

^1^ (S) indicates strain is susceptible to phage infection, (I) indicates strain has an intermediate phenotype and is only somewhat susceptible at a reduced MOI, and (R) indicates that the strain is resistant to phage infection.

**Table 3:** Summary of key genomic features of Atu_ph04 and Atu_ph08.

Phage	Genome Length (bp)	G + C content (%)	Number of ORFs	Number of Hypothetical Proteins	Number of ORFs with Predicted Functions	Number of ORFans	Number of tRNAs
Atu_ph04	143,349	49.4	223	67	73	83	1
Atu_ph08	59,034	59.7	75	43	32	3	0

## Supplementary Materials

The following are available online at https://www.mdpi.com/1999-4915/11/6/528/s1, Supplementary Figure S1: Restriction fragment analysis of digested Atu_ph04 genomic DNA. Supplementary Figure S2: Analysis of DarB-like protein in Atu_ph08. Supplementary Table S1: Atu_ph04 genes organized by predicted function. Supplementary Table S2: Comparative analysis of Atu_ph04 gene products with related phages. Supplementary Table S3: T4 core proteins found in Atu_ph04. Supplementary Table S4: Atu_ph08 genes organized by predicted function. Supplementary Table S5: Comparative analysis of Atu_ph08 gene products with related phages. Supplementary Table S6: Atu_ph08 gene products present in other *Agrobacterium* phages.
