# Supplementary material for: Isolation and Characterization T4- and T7-Like Phages that Infect the Bacterial Plant Pathogen Agrobacterium tumefaciens
Source: Viruses. 2019 Jun 7;11(6):528. doi: 10.3390/v11060528 (PMC6630229; doi:10.3390/v11060528)
Supplement: Supplementary file 1 [file viruses-11-00528-s001.pdf]

Supplementary Materials

Isolation and Characterization T4- and T7-like Phages that Infect the Bacterial Plant Pathogen, *Agrobacterium tumefaciens*

Hedieh Attai † and Pamela J.B. Brown *

Division of Biological Sciences, University of Missouri, Columbia, MO 65211 USA
†Present address: Department of Pathology, University of California at San Diego, La Jolla, CA92093; hattai@ucsd.edu
*Correspondence: brownpb@missouri.edu; Tel.: (+1-573-884-0214)

SUPPLEMENTARY FIGURES

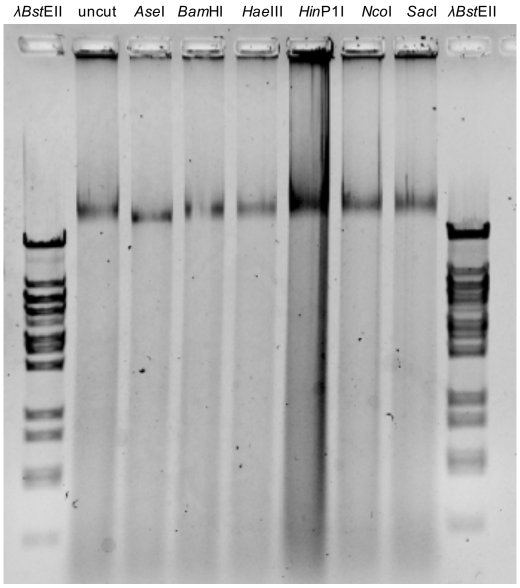

Supplementary Figure S1: Restriction fragment analysis of digested Atu_ph04 genomic DNA. DNA is loaded onto a 0.7% agarose gel.

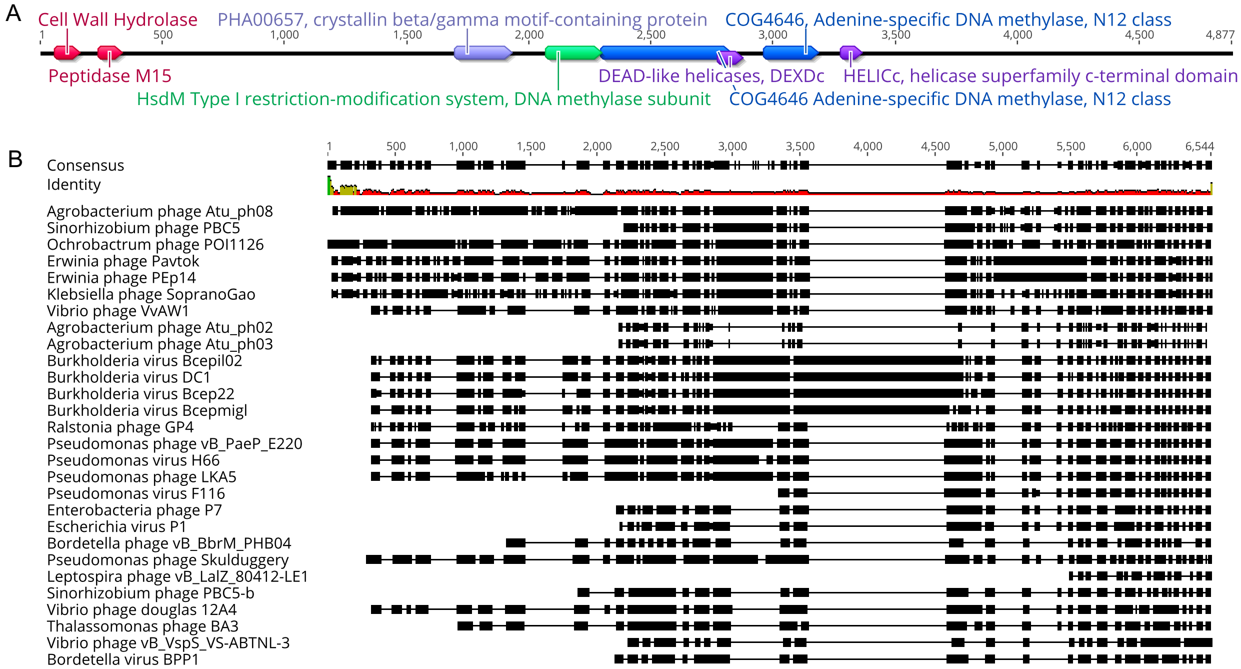

Supplementary Figure S2: Analysis of DarB-like protein in Atu_ph08. (A) Domain structure of DarB-like protein in Atu_ph08. (B) ClustalW alignment of DarB-like proteins in other T7-like phages. Dark black blocks indicate regions of synteny.

SUPPLEMENTARY TABLES

Supplementary Table S1: Atu_ph04 genes organized by predicted function.

CDS #	RAST annotated function	Updated assigned function	Length (bp)	Category
1	hypothetical protein CDS	putative T7-like tail fiber protein	186	Structural
2	hypothetical protein CDS		600	Hypothetical
3	hypothetical protein CDS		321	Hypothetical
4	hypothetical protein CDS		246	Hypothetical
5	hypothetical protein CDS		210	Hypothetical
6	UDP-galactopyranose mutase (EC 5.4.99.9) CDS		1173	Other/Bacterial
7	hypothetical protein CDS		315	Hypothetical
8	hypothetical protein CDS		159	Hypothetical
9	hypothetical protein CDS		354	Hypothetical
10	N-acetylmuramoyl-L-alanine amidase (EC 3.5.1.28) CDS		942	Lysis
11	hypothetical protein CDS		216	Hypothetical
12	hypothetical protein CDS		516	Hypothetical
13	hypothetical protein CDS		366	Hypothetical
14	Phage protein CDS		351	Hypothetical
15	hypothetical protein CDS		399	Hypothetical
16	hypothetical protein CDS		213	Hypothetical
17	Phosphohydrolase (MutT/nudix family protein) CDS		588	Nucleotide metabolism
18	hypothetical protein CDS		600	Hypothetical
19	hypothetical protein CDS		249	Hypothetical
20	hypothetical protein CDS		309	Hypothetical
21	hypothetical protein CDS		546	Hypothetical
22	hypothetical protein CDS		198	Hypothetical
23	hypothetical protein CDS	putative glutaredoxin	282	Nucleotide metabolism
24	Ribonucleotide reductase of class Ia (aerobic), alpha subunit (EC 1.17.4.1) CDS		1662	Nucleotide metabolism

25	Ribonucleotide reductase of class Ia (aerobic), beta subunit (EC 1.17.4.1) CDS		1053	Nucleotide metabolism
26	Phage-associated DNA primase (EC 2.7.7.-) #gp61 CDS		1041	DNA replication
27	hypothetical protein CDS		339	Hypothetical
28	hypothetical protein CDS		402	Hypothetical
29	hypothetical protein CDS		354	Hypothetical
30	hypothetical protein CDS		297	Hypothetical
31	hypothetical protein CDS		510	Hypothetical
32	hypothetical protein CDS		480	Hypothetical
33	hypothetical protein CDS	putative uracil DNA glycosylase	468	DNA repair
34	hypothetical protein CDS		273	Hypothetical
35	hypothetical protein CDS		405	Hypothetical
36	hypothetical protein CDS		255	Hypothetical
37	hypothetical protein CDS		219	Hypothetical
38	hypothetical protein CDS		291	Hypothetical
39	hypothetical protein CDS		207	Hypothetical
40	Phage DNA end protector during packaging CDS		627	DNA associated
41	hypothetical protein CDS	putative baseplate tail tube initiator	537	Structural
42	Phage baseplate hub subunit # T4-like gp26 CDS		702	Structural
43	hypothetical protein CDS	putative baseplate hub assembly catalyst	177	Structural
44	hypothetical protein CDS		381	Hypothetical
45	hypothetical protein CDS		1812	Hypothetical
46	hypothetical protein CDS		273	Hypothetical
47	Single stranded DNA-binding protein, phage-associated CDS		1011	DNA replication
48	hypothetical protein CDS		555	Hypothetical
49	hypothetical protein CDS		312	Hypothetical

50	hypothetical protein CDS		288	Hypothetical
51	hypothetical protein CDS		540	Hypothetical
52	hypothetical protein CDS	putative homing endonuclease	654	DNA replication
53	Phage terminase, large subunit #T4-like phage Gp17 CDS		1734	Structural
54	T4-like phage baseplate hub + tail lysozyme CDS		2562	Structural
55	hypothetical protein CDS		126	Hypothetical
56	hypothetical protein CDS		285	Hypothetical
57	hypothetical protein CDS		507	Hypothetical
58	T4-like phage protein, T4 GC1630 CDS	putative homing endonuclease	459	DNA replication
59	probable ATP-dependent helicase CDS		1614	DNA replication
60	Phage endonuclease CDS	denV endonuclease V, N-glycosylase UV repair enzyme	318	DNA repair
61	hypothetical protein CDS		264	Hypothetical
62	hypothetical protein CDS		414	Hypothetical
63	Phage ribonuclease H (EC 3.1.26.4) #T4-like phage Rnh #T4 GC0870 CDS		909	DNA replication
64	hypothetical protein CDS		720	Hypothetical
65	hypothetical protein CDS		438	Hypothetical
66	hypothetical protein CDS	tail completion and sheath stabilizer protein	522	Structural
67	Single stranded DNA-binding protein, phage-associated #T4-like phage Gp32 CDS		486	DNA replication
68	hypothetical protein CDS	putative homing endonuclease	765	DNA replication
69	Phage head completion protein CDS		459	Structural

70	Phage tail sheath CDS		2517	Structural
71	hypothetical protein CDS	putative tail tube monomer	627	Structural
72	Phage portal vertex of the head #T4-like phage Gp20 CDS		1575	Structural
73	hypothetical protein CDS		153	Hypothetical
74	Phage prohead core protein CDS		666	Structural
75	MJ0042 family finger-like protein CDS		1131	Hypothetical
76	Phage major capsid protein of Caudovirales CDS		1332	Structural
77	hypothetical protein CDS		183	Hypothetical
78	DNA helicase, phage-associated CDS		1503	DNA replication
79	hypothetical protein CDS		324	Hypothetical
80	hypothetical protein CDS	putative terminase DNA packaging enzyme small subunit	504	DNA associated
81	hypothetical protein CDS		186	Hypothetical
82	hypothetical protein CDS	putative base plate wedge subunit	387	Structural
83	gp6 baseplate wedge subunit CDS		1782	Structural
84	hypothetical protein CDS	baseplate wedge subunit-like protein	3114	Structural
85	hypothetical protein CDS		1227	Hypothetical
86	Phage protein CDS		1545	Hypothetical
87	Phosphate starvation-inducible protein PhoH, predicted ATPase CDS		783	Other/Bacterial
88	hypothetical protein CDS		348	Hypothetical
89	RNA polymerase ECF-type sigma factor CDS		483	Transcription
90	hypothetical protein CDS		294	Hypothetical

91	hypothetical protein CDS		345	Hypothetical
92	hypothetical protein CDS		150	Hypothetical
93	hypothetical protein CDS	putative baseplate tail tube cap	780	Structural
94	hypothetical protein CDS	putative base plate wedge component	579	Structural
95	hypothetical protein CDS		414	Hypothetical
96	Phage recombination protein CDS		1164	DNA associated
97	hypothetical protein CDS	DNA primase/helicase	1434	DNA replication
98	hypothetical protein CDS	putative holliday junction resolvase	624	DNA associated
99	hypothetical protein CDS		435	Hypothetical
100	hypothetical protein CDS	5'-deoxynucleotidase	621	DNA associated
101	hypothetical protein CDS	putative deoxynucleotide monophosphate kinase	786	DNA associated
102	hypothetical protein CDS		363	Hypothetical
103	hypothetical protein CDS		465	Hypothetical
104	Deoxycytidylate 5-hydroxymethyltransferase (EC 2.1.2.8) CDS		744	DNA modification
105	hypothetical protein CDS		1017	Hypothetical
106	hypothetical protein CDS		642	Hypothetical
107	hypothetical protein CDS		594	Hypothetical
108	hypothetical protein CDS		423	Hypothetical
109	hypothetical protein CDS		378	Hypothetical
110	Phage DNA topoisomerase large subunit (EC 5.99.1.3) #T4-like gp60 #T4 GC1464 CDS		1938	DNA replication
111	hypothetical protein CDS		306	Hypothetical
112	hypothetical protein CDS		552	Hypothetical

113	Topoisomerase IV subunit A (EC 5.99.1.-) CDS		1365	DNA replication
114	hypothetical protein CDS		120	Hypothetical
115	hypothetical protein CDS		336	Hypothetical
116	COG1896: Predicted hydrolases of HD superfamily CDS		723	Lysis
117	hypothetical protein CDS		336	Hypothetical
118	hypothetical protein CDS		360	Hypothetical
119	hypothetical protein CDS	sigma factor for late transcription	513	Transcription
120	hypothetical protein CDS	recombination endonuclease subunit	951	DNA associated
121	Phage recombination-related endonuclease Gp46 CDS		1647	DNA associated
122	hypothetical protein CDS	sliding clamp DNA polymerase accessory protein	729	DNA replication
123	Replication factor C small subunit CDS	Sliding clamp loader subunit	948	DNA replication
124	hypothetical protein CDS	putative clamp loader subunit	405	DNA replication
125	hypothetical protein CDS		177	Hypothetical
126	hypothetical protein CDS		159	Hypothetical
127	hypothetical protein CDS		267	Hypothetical
128	hypothetical protein CDS		690	Hypothetical
129	hypothetical protein CDS		258	Hypothetical
130	hypothetical protein CDS		381	Hypothetical
131	hypothetical protein CDS		210	Hypothetical
132	hypothetical protein CDS		234	Hypothetical
133	DNA polymerase (EC 2.7.7.7), phage-associated #T4-like phage gp43 #T4 GC0178 CDS		2640	DNA replication
134	hypothetical protein CDS		768	Hypothetical
135	hypothetical protein CDS		120	Hypothetical

136	hypothetical protein CDS		303	Hypothetical
137	hypothetical protein CDS		546	Hypothetical
138	DNA ligase, phage-associated CDS		1257	DNA replication
139	hypothetical protein CDS		336	Hypothetical
140	hypothetical protein CDS		213	Hypothetical
141	hypothetical protein CDS		189	Hypothetical
142	hypothetical protein CDS		201	Hypothetical
143	hypothetical protein CDS		594	Hypothetical
144	hypothetical protein CDS		165	Hypothetical
145	Phage protein CDS	predicted alternative thymidylate synthase	984	Nucleotide metabolism
146	hypothetical protein CDS		282	Hypothetical
147	hypothetical protein CDS		165	Hypothetical
148	hypothetical protein CDS	putative GT1 glycosyltransferase protein	1164	Nucleotide metabolism
149	hypothetical protein CDS		405	Hypothetical
150	FIG00451076: hypothetical protein CDS		255	Hypothetical
151	hypothetical protein CDS	putative homing endonuclease	714	DNA associated
152	hypothetical protein CDS	putative ParB-like nuclease domain containing protein	450	DNA associated
153	hypothetical protein CDS		474	Hypothetical
154	hypothetical protein CDS		216	Hypothetical
155	hypothetical protein CDS		303	Hypothetical
156	hypothetical protein CDS		303	Hypothetical
157	hypothetical protein CDS		267	Hypothetical
158	hypothetical protein CDS		183	Hypothetical
159	hypothetical protein CDS		246	Hypothetical
160	hypothetical protein CDS		501	Hypothetical
161	hypothetical protein CDS		156	Hypothetical
162	hypothetical protein CDS		357	Hypothetical

163	Glycine-rich cell wall structural protein 1.8 precursor CDS		657	Other/Bacterial
164	hypothetical protein CDS		480	Hypothetical
165	hypothetical protein CDS		489	Hypothetical
166	hypothetical protein CDS		279	Hypothetical
167	hypothetical protein CDS		555	Hypothetical
168	hypothetical protein CDS		135	Hypothetical
169	hypothetical protein CDS	DNA repair exonuclease	489	DNA repair
170	hypothetical protein CDS		267	Hypothetical
171	hypothetical protein CDS		207	Hypothetical
172	hypothetical protein CDS		195	Hypothetical
173	hypothetical protein CDS		198	Hypothetical
174	hypothetical protein CDS		201	Hypothetical
175	hypothetical protein CDS		417	Hypothetical
176	hypothetical protein CDS		363	Hypothetical
177	hypothetical protein CDS		300	Hypothetical
178	hypothetical protein CDS		198	Hypothetical
179	hypothetical protein CDS		225	Hypothetical
180	hypothetical protein CDS	von Willebrand factor type A domain containing protein	702	Other/Bacterial
181	hypothetical protein CDS		225	Hypothetical
182	hypothetical protein CDS		303	Hypothetical
183	hypothetical protein CDS		207	Hypothetical
184	hypothetical protein CDS		276	Hypothetical
185	Polymerase epsilon subunit CDS		858	DNA associated
186	hypothetical protein CDS		285	Hypothetical
187	hypothetical protein CDS		291	Hypothetical
188	hypothetical protein CDS		264	Hypothetical
189	dCMP deaminase (EC 3.5.4.12); Late competence protein ComEB CDS		600	DNA associated
190	hypothetical protein CDS		510	Hypothetical

191	hypothetical protein CDS		210	Hypothetical
192	hypothetical protein CDS		234	Hypothetical
193	hypothetical protein CDS		462	Hypothetical
194	Phage protein CDS	putative exonuclease	825	DNA associated
195	hypothetical protein CDS	DNA primase	531	DNA replication
196	hypothetical protein CDS		282	Hypothetical
197	hypothetical protein CDS		363	Hypothetical
198	hypothetical protein CDS		372	Hypothetical
199	hypothetical protein CDS		231	Hypothetical
200	hypothetical protein CDS		234	Hypothetical
201	hypothetical protein CDS		261	Hypothetical
202	hypothetical protein CDS		423	Hypothetical
203	hypothetical protein CDS		300	Hypothetical
204	hypothetical protein CDS		330	Hypothetical
205	hypothetical protein CDS		744	Hypothetical
206	hypothetical protein CDS		609	Hypothetical
207	hypothetical protein CDS		231	Hypothetical
208	hypothetical protein CDS		201	Hypothetical
209	hypothetical protein CDS		435	Hypothetical
210	hypothetical protein CDS		510	Hypothetical
211	hypothetical protein CDS		579	Hypothetical
212	hypothetical protein CDS		1347	Hypothetical
213	T4-like phage baseplate hub + tail lysozyme CDS		876	Structural
214	hypothetical protein CDS		297	Hypothetical
215	Phage neck protein #Gp13 CDS		756	Structural
216	Gp14 neck protein CDS		843	Structural
217	hypothetical protein CDS		360	Hypothetical
218	Phage tail assembly CDS		882	Structural
219	hypothetical protein CDS	baseplate wedge	1461	Structural
220	Phage virulence-associated VriC protein CDS	VrlC protein	5088	Structural
221	hypothetical protein CDS		207	Hypothetical
222	Phage tail fibers CDS		1791	Structural

223	FIG00920814: hypothetical protein CDS		1149	Hypothetical

Supplementary Table S2: Comparative analysis of Atu_ph04 gene products with related phages.

gp #	Functional annotation	vB_RleM_P10VF	phiM9	Cr30	syn9	syn30	syn33	T4	Atu_ph07	phiM12	Ccr Colossus	phiN3	KVP 40	Melville
6	UDP-galactopyranose mutase	3.00E-125	1.00E-128											
9	hypothetical	9.00E-22												
10	N-acetylmuramoyl-L-alanine amidase	4.00E-90	1.00E-87											
11	hypothetical	6.00E-12	5.00E-14											
12	hypothetical	1.00E-31	2.00E-38											
13	hypothetical		4.00E-27											
14	hypothetical	1.00E-26	1.00E-21											
15	hypothetical	9.00E-17								4.00E-11				
17	Phosphohydrolase (MutT/nudix family protein)			7.00E-16						5.00E-23	9.00E-24	6.00E-23		
20	hypothetical										2.00E-11			
23	hypothetical	2.00E-23	1.00E-20											
24	Ribonucleotide reductase of class Ia (aerobic), alpha subunit	0	0		3.00E-17	2.00E-16	2.00E-16	9.00E-17	0		5.00E-70		2.00 E-17	1.00E-16
25	Ribonucleotide reductase of class Ia (aerobic), beta subunit	4.00E-156	1.00E-162						3.00E-104					

26	Phage-associated DNA primase	3.00E-87	2.00E-108		4.00E-42	1.00E-41	8.00E-34	2.00E-37		1.00E-37		8.00E-37	7.00E-22	9.00E-34
27	hypothetical	1.00E-32	1.00E-43											
28	hypothetical	6.00E-29	3.00E-36											
29	hypothetical	9.00E-10	1.00E-23											
30	hypothetical	3.00E-24	4.00E-21											
33	hypothetical	1.00E-17	1.00E-19											
36	hypothetical	1.00E-06												
40	Phage DNA end protector during packaging	3.00E-102	3.00E-100	2.00E-17				6.00E-29		2.00E-25				3.00E-28
41	putative baseplate tail tube initiator	7.00E-56	3.00E-67		3.00E-06		6.00E-08						2.00E-08	
42	Phage baseplate hub subunit	1.00E-80	3.00E-84										3.00E-05	9.00E-07
43	hypothetical	3.00E-18	5.00E-13	3.00E-04										
44	hypothetical	8.00E-17	2.00E-19											
45	hypothetical	2.00E-122	1.00E-141											
46	hypothetical		7.00E-07											
47	Single stranded DNA-binding protein	6.00E-125	4.00E-105	1.00E-25		3.00E-26	7.00E-28			3.00E-24		2.00E-24	5.00E-16	
48	hypothetical	7.00E-12	2.00E-16											
49	hypothetical	5.00E-20	7.00E-14											
50	hypothetical	5.00E-16	1.00E-14											

51	hypothetical	4.00E-66	9.00E-70											
52	hypothetical	5.00E-22	2.00E-36	6.00E-10				2.00E-13		2.00E-07		3.00E-07		
53	Phage terminase, large subunit	0	0	2.00E-114	9.00E-114	7.00E-117	1.00E-112	2.00E-105	3.00E-33	5.00E-120		8.00E-120	2.00E-99	2.00E-108
54	T4-like phage baseplate hub + tail lysozyme	0	0	8.00E-16				6.00E-14		6.00E-18		2.00E-17	7.00E-20	7.00E-16
58	putative homing endonuclease	4.00E-79	2.00E-66							3.00E-72	6.00E-60	2.00E-71	2.00E-57	7.00E-60
59	probable ATP-dependent helicase	0	0											
60	Phage endonuclease	1.00E-31	4.00E-37					2.00E-18					2.00E-07	
62	hypothetical	8.00E-36	2.00E-32						7.00E-20		1.00E-24			
63	Phage ribonuclease H	2.00E-135	8.00E-140	1.00E-28						3.00E-32				
64	hypothetical	6.00E-53	3.00E-56											
65	hypothetical	9.00E-16	2.00E-27											
66	tail completion and sheath stabilizer protein	3.00E-55	9.00E-55					2.00E-08						7.00E-12
67	Single stranded DNA-binding protein	4.00E-52	5.00E-48			1.00E-14	3.00E-14	3.00E-08		9.00E-15				1.00E-11
68	hypothetical		2.00E-23											

69	Phage head completion protein	1.00E-76	2.00E-77	9.00E-08	5.00E-36	6.00E-35	6.00E-37	5.00E-34	1.00E-25	4.00E-36			2.00E-40	1.00E-41
70	Phage tail sheath	0	0	6.00E-47	2.00E-46	1.00E-43	4.00E-42	3.00E-31		4.00E-61		3.00E-61	3.00E-34	2.00E-33
71	putative tail tube monomer	5.00E-87	2.00E-98		3.00E-14	2.00E-16	7.00E-18							
72	Phage portal vertex of the head	0	0	9.00E-98	3.00E-109	7.00E-113	3.00E-76	5.00E-97	7.00E-19	3.00E-87		4.00E-87	4.00E-86	3.00E-93
74	Phage prohead core protein	7.00E-93	7.00E-94	3.00E-26	8.00E-34	5.00E-34	3.00E-33	1.00E-14		3.00E-38		3.00E-38	2.00E-30	2.00E-16
75	MJ0042 family finger-like protein	2.00E-52	4.00E-55											
76	Phage major capsid protein	0	0	2.00E-71	1.00E-83	4.00E-81	9.00E-85	5.00E-48	5.00E-31	1.00E-92		1.00E-92	2.00E-55	3.00E-48
78	DNA helicase	0	0	2.00E-78	1.00E-68	3.00E-66	7.00E-65	2.00E-69	3.00E-18	2.00E-67		4.00E-66	2.00E-72	1.00E-71
80	putative terminase DNA packaging enzyme small subunit	6.00E-48	2.00E-43											
82	putative base plate wedge subunit	5.00E-58	2.00E-57											
83	baseplate wedge subunit	0	0	2.00E-54	5.00E-46	2.00E-46	1.00E-43			1.00E-55		2.00E-56		
84	baseplate wedge subunit-like protein	0	0							5.00E-06		5.00E-06		
85	hypothetical		0.001											

87	Phosphate starvation-inducible protein PhoH, predicted ATPase	2.00E-99	2.00E-111	1.00E-23	1.00E-21	2.00E-21	1.00E-22		2.00E-54		8.00E-29	1.00E-17	3.00E-29	
92	hypothetical	1.00E-05												
93	putative baseplate tail tube cap	5.00E-123	5.00E-135											
94	putative base plate wedge component	2.00E-60	4.00E-66											
95	hypothetical	2.00E-28	2.00E-27											
96	Phage recombination protein	0	0	7.00E-55				2.00E-71	5.00E-17	8.00E-65		5.00E-65	5.00E-72	5.00E-71
97	DNA primase/helicase	0	0	6.00E-79	4.00E-83	5.00E-84	1.00E-84	2.00E-56	1.00E-27	9.00E-76		1.00E-74	1.00E-68	2.00E-56
98	putative holliday junction resolvase	3.00E-63	8.00E-51	1.00E-13										
100	hypothetical	1.00E-70	1.00E-59											
101	putative deoxynucleotide monophosphate kinase	6.00E-58	4.00E-66											
103	hypothetical	4.00E-71	3.00E-65											
104	Deoxycytidylate 5-hydroxymethyltransferase	9.00E-79	2.00E-85											
105	hypothetical	5.00E-89	9.00E-91											

106	hypothetical	3.00E-10	7.00E-20											
107	hypothetical			3.00E-09			8.00E-16							
109	hypothetical	1.00E-28												
110	Phage DNA topoisomerase large subunit	0	0					5.00E-26	3.00E-146				2.00E-49	5.00E-53
113	Topoisomerase IV subunit A	0	0					8.00E-33	1.00E-98				2.00E-37	3.00E-31
119	sigma factor for late transcription	1.00E-47	2.00E-51											
120	recombination endonuclease subunit	1.00E-67	4.00E-70											
121	Phage recombination-related endonuclease	6.00E-164	2.00E-173	9.00E-56	7.00E-59	1.00E-59	5.00E-62	6.00E-65		4.00E-59		8.00E-59	5.00E-28	1.00E-59
122	sliding clamp DNA polymerase accessory protein	6.00E-58	8.00E-70											
123	Replication factor C small subunit	5.00E-128	4.00E-117					7.00E-05	2.00E-31				7.00E-51	6.00E-04
124	putative clamp loader subunit	4.00E-50	4.00E-48	3.00E-09	5.00E-05	3.00E-05								
129	hypothetical	2.00E-36	2.00E-30											
131	hypothetical		2.00E-05											

133	DNA polymerase (EC 2.7.7.7), phage-associated	0	0	3.00E-89	2.00E-109	6.00E-103	1.00E-105	2.00E-81	8.00E-23	8.00E-104		9.00E-104	6.00 E-74	2.00E-80
134	hypothetical	2.00E-90	5.00E-102											
138	DNA ligase, phage-associated	8.00E-145	1.00E-153										6.00 E-26	
139	hypothetical	2.00E-21	3.00E-14											
143	hypothetical	1.00E-43	7.00E-38										6.00 E-13	
145	predicted alternative thymidylate synthase										3.00E-39			
148	putative GT1 glycosyltransferase protein	2.00E-127	1.00E-118											
150	FIG00451076: hypothetical protein	7.00E-22	8.00E-20	1.00E-22										
151	putative homing endonuclease		4.00E-41											
152	putative ParB-like nuclease domain containing protein	1.00E-22	5.00E-19											
153	hypothetical	3.00E-61	2.00E-59											
157	hypothetical	2.00E-08												
159	hypothetical	4.00E-12	3.00E-13							3.00E-23				
160	hypothetical	8.00E-07												

164	hypothetical	7.00E-13	2.00E-23											
165	hypothetical	3.00E-30	4.00E-37											
166	hypothetical		0.001						2.00E-04					
167	hypothetical	1.00E-09												
168	hypothetical	4.00E-04												
169	DNA repair exonuclease	1.00E-25	2.00E-33						3.00E-16	2.00E-31		1.00E-31		
180	von Willebrand factor type A domain containing protein	1.00E-81												
182	hypothetical								6.00E-16	7.00E-27			4.00 E-04	
185	Polymerase epsilon subunit	7.00E-112	3.00E-112						7.00E-28					
188	hypothetical	5.00E-14												
189	dCMP deaminase (EC 3.5.4.12); Late competence protein ComEB	1.00E-49	1.00E-49					9.00E-10					3.00 E-14	4.00E-11
190	hypothetical	2.00E-09	1.00E-10											
192	hypothetical	0.001	7.00E-04											
194	putative exonuclease	7.00E-112	1.00E-100	1.00E-26	6.00E-22	5.00E-21	1.00E-21		2.00E-14			3.00E-22		
201	hypothetical	2.00E-07	7.00E-07											
202	hypothetical	3.00E-27												
206	hypothetical	6.00E-14							2.00E-12					

207	hypothetical	2.00E-06												
209	hypothetical	2.00E-11	3.00E-20											
212	hypothetical	0	0				2.00E-06							
213	T4-like phage baseplate hub + tail lysozyme	3.00E-147	3.00E-141	2.00E-18	8.00E-13	3.00E-13	4.00E-14	3.00E-15		4.00E-12		4.00E-12		9.00E-16
215	Phage neck protein	4.00E-106	3.00E-106	3.00E-25	4.00E-24	7.00E-20		7.00E-09		5.00E-24		2.00E-24	5.00E-04	4.00E-06
216	Gp14 neck protein	3.00E-122	7.00E-109	1.00E-14	1.00E-08					2.00E-15		3.00E-15		
217	hypothetical	8.00E-37	6.00E-31											
218	Phage tail assembly	1.00E-82	3.00E-106	7.00E-24	9.00E-24	6.00E-19	2.00E-26			2.00E-18			5.00E-14	
219	baseplate wedge	4.00E-147	2.00E-170	7.00E-09										
220	Phage virulence-associated VriC protein	0	0	3.00E-28	6.00E-41	4.00E-43	2.00E-42			9.00E-69		4.00E-67		
221	hypothetical		7.00E-17	9.00E-05										
222	Phage tail fibers	8.00E-175	2.00E-167											2.00E-06
223	FIG00920814: hypothetical protein	5.00E-66	7.00E-76											
	TOTAL	115	109	32	23	23	24	25	22	30	7	25	29	26

Supplementary Table S3: T4 core proteins found in Atu_ph04.

T4 protein	T4 protein function	Match in Atu_ph04*	Identity (%)	E-value	Query cover (%)	Atu_ph04 protein name	Atu_ph04 gp #
**Phage morphogenesis**							
gp4	head completion protein	yes	39	1e-35	96	Head completion protein	69
gp5	baseplate lysozyme hub component	yes	45	2e-16	17	T4-like phage baseplate hub + tail lysozyme	213
			30	1e-15	32	T4-like phage baseplate hub + tail lysozyme	54
gp13	head completion protein	unresolved	21	6e-08	99	Neck protein	215
gp15	tail completion protein	yes	25	1e-11	74	Phage tail assembly	218
gp17	subunit of the terminase for DNA packaging	yes	37	7e-107	81	Terminase large subunit	53
gp18	tail tube subunit	yes	28	9e-32	91	Tail sheath	70
gp20	head portal vertex protein	yes	36	2e-98	85	Portal vertex of the head	72
gp21	prohead core protein and protease	yes	33	6e-16	66	Prohead core protein	74
gp22	prohead core protein	no					
gp23	precursor of major head protein	yes	34	4e-17	96	Major capsid protein	76
gp25	base plate wedge subunit	no					
gp34	proximal tail fiber protein subunit	no					
gp36	small distal tail fiber protein subunit	no					
**DNA replication, repair, and recombination**							

gp43	DNA polymerase	yes	28	7e-83	97	DNA polymerase	133
gp44	sliding clamp loader complex tetramer	yes	32	6e-47	99	Replication factor C small subunit	123
gp41	helicase-primer complex hexamer	no					
gp46	subunit of a recombination nuclease complex	yes	29	2e-66	99	Phage recombination-related endonuclease	121
gp47	subunit of a recombination nuclease complex	no					
UvsW	recombination DNA-RNA helicase, DNA-dependent ATPase	yes	33	8e-71	82	DNA helicase	78
**Auxillary metabolism**							
nrdA	subunit of an aerobic ribonucleotide reductase complex	yes	23	4e-18	58	Ribonucleotide reductase of 1a (aerobic), alpha subunit	24
nrdB	subunit of an aerobic ribonucleotide reductase complex	no					
**Gene expression**							
gp55	sigma factor for late transcription	no					

*Atu_ph04 matches with E-values above 1E-10 are considered “yes” matches and those between 1E-10 and 1E-03 are “unresolved.” Matches with E-values lower than 1E-03 were not considered significant.

Supplementary Table S4: Atu_ph08 genes organized by predicted function.

CDS #	RAST annotated function	Updated assigned function	Length (bp)	Category
1	Phage protein CDS	XRE transcriptional regulator	300	Transcription
2	hypothetical protein CDS		297	Hypothetical
3	Phage protein CDS		417	Hypothetical
4	FIG00451076: hypothetical protein CDS	DUF2312 domain-containing protein	396	Hypothetical
5	hypothetical protein CDS	GcrA cell cycle regulator	588	Transcription
6	hypothetical protein CDS		495	Hypothetical
7	C-5 cytosine-specific DNA methylase CDS		1866	DNA modification
8	hypothetical protein CDS		351	Hypothetical
9	hypothetical protein CDS	putative PRK12775-containing protein	246	Posttranslational modification
10	hypothetical protein CDS	NERD domain-containing protein	741	DNA processing
11	Phage protein CDS		627	Hypothetical
12	Phage protein CDS		804	Hypothetical
13	probable terminase large subunit CDS		1557	Structural
14	Phage protein CDS		429	Hypothetical
15	Phage portal protein CDS		2232	Structural
16	Phage protein CDS		1011	Hypothetical
17	hypothetical protein CDS		195	Hypothetical
18	hypothetical protein CDS		294	Hypothetical
19	hypothetical protein CDS		477	Hypothetical

20	Methyl-accepting chemotaxis protein I (serine chemoreceptor protein) CDS		786	Other/Bacterial
21	helicase, Snf2 family CDS	Adenine-specific DNA methylase, N12 class; DarB-like protein	14634	DNA modification
22	Phage protein CDS		1311	Hypothetical
23	Phage protein CDS	tail fiber domain-containing protein	1161	Structural
24	Phage protein CDS	N-acetyltransferase	450	DNA modification
25	hypothetical protein CDS		246	Hypothetical
26	Phage protein CDS	putative virion structural protein	1740	Structural
27	hypothetical protein CDS	DUF4376 domain-containing protein	603	Hypothetical
28	Phage tail fibers CDS		1074	Structural
29	Phage protein CDS		279	Hypothetical
30	hypothetical protein CDS		312	Hypothetical
31	Phage protein CDS	major capsid protein	417	Structural
32	Phage protein CDS	DUF4238 domain-containing protein	627	Hypothetical
33	Phage protein CDS		702	Hypothetical
34	hypothetical protein CDS		363	Hypothetical
35	Phage protein CDS		462	Hypothetical
36	Phage protein CDS	N4-gp56 family major capsid protein	1131	Structural
37	protein of unknown function DUF847 CDS	secretion activator protein; lysozyme-like protein	753	Lysis
38	hypothetical protein CDS	Holin of 3TMs, for gene-transfer release	525	Lysis
39	Phage protein CDS		333	Hypothetical

40	hypothetical protein CDS	AlpA family phage regulatory protein	231	Transcription
41	Integrase CDS		1299	DNA recombination
42	hypothetical protein CDS	Arc family DNA-binding protein	192	DNA-associated
43	hypothetical protein CDS		402	Hypothetical
44	hypothetical protein CDS		498	Hypothetical
45	hypothetical protein CDS		189	Hypothetical
46	hypothetical protein CDS	DUF551 domain-containing protein	186	Hypothetical
47	hypothetical protein CDS		324	Hypothetical
48	hypothetical protein CDS		219	Hypothetical
49	hypothetical protein CDS	3'-5' exoribonuclease	705	DNA-associated
50	hypothetical protein CDS		264	Hypothetical
51	hypothetical protein CDS		645	Hypothetical
52	Phage protein CDS		330	Hypothetical
53	Phage DNA modification methyltransferase CDS		753	DNA modification
54	hypothetical protein CDS		219	Hypothetical
55	Bacteriophage protein gp37 CDS	DUF5131 family protein	921	Hypothetical
56	COG1896: Predicted hydrolases of HD superfamily CDS	metal-dependent phosphohydrolase	606	DNA modification
57	Phage protein CDS		687	Hypothetical

58	hypothetical protein CDS		426	Hypothetical
59	Phage-related protein CDS	morphogenetic protein	708	Structural
60	Phage protein CDS		399	Hypothetical
61	hypothetical protein CDS		231	Hypothetical
62	hypothetical protein CDS		525	Hypothetical
63	hypothetical protein CDS		159	Hypothetical
64	Phage protein CDS	C4-dicarboxylate ABC transporter substrate-binding protein	882	Other/Bacterial
65	Transcriptional regulator CDS	C2-like repressor protein	417	Transcription
66	hypothetical protein CDS		219	Hypothetical
67	hypothetical protein CDS	class I SAM-dependent methyltransferase	582	DNA modification
68	hypothetical protein CDS		288	Hypothetical
69	Phage protein CDS		309	Hypothetical
70	hypothetical protein CDS		123	Hypothetical
71	Predicted periplasmic protein CDS	Clp protease	651	Posttranslational modification
72	hypothetical protein CDS	XRE family transcriptional regulator	306	Transcription
73	hypothetical protein CDS		240	Hypothetical
74	hypothetical protein CDS		243	Hypothetical
75	hypothetical protein CDS		207	Hypothetical

Supplementary Table S5: Comparative analysis of Atu_ph08 gene products with related phages.

gp #	Functional annotation	PBC5	POI1126	Soprano Gao	Pavtok	DC1	Bcep22	PS-1	phiKMV	T7	Atu_ph03
1	XRE transcriptional regulator	7.00E-44									
3	Hypothetical	4.00E-26	1.00E-10								
4	Hypothetical	1.00E-38	5.00E-20								
10	NERD domain-containing protein		1.00E-32								
11	Hypothetical	8.00E-66									
12	Hypothetical	1.00E-77									
13	probable terminase large subunit	0	0	1.00E-143	5.00E-145	2.00E-127	1.00E-127	2.00E-100			
14	Hypothetical	6.00E-72	2.00E-40	3.00E-17	1.00E-17		9.00E-15				
15	Phage portal protein	0	0	0	0	0	0				
16	Hypothetical	7.00E-79	6.00E-07	9.00E-11	2.00E-18	4.00E-18	2.00E-19				
17	Hypothetical					6.00E-05					
21	Adenine-specific DNA methylase, N12 class; helicase, Snf2 family	0	0	0	0	0	0				5.00E-48
22	Hypothetical	1.00E-106	2.00E-45	5.00E-08	2.00E-20						
23	tail fiber domain-containing protein	0	4.00E-160	1.00E-110	2.00E-114	1.00E-75	3.00E-73				
24	N-acetyltransferase	3.00E-84	2.00E-43	9.00E-36	1.00E-35	3.00E-41	1.00E-39				
25	Hypothetical		2.00E-13			5.00E-08	2.00E-08				
26	putative virion structural protein	0	0	2.00E-140	1.00E-148	0.00E+00	1.00E-179				
28	Phage tail fibers	2.00E-31	3.00E-28	9.00E-23	4.00E-17	1.00E-26	2.00E-26				
29	Hypothetical	7.00E-38	1.00E-31			2.00E-18	3.00E-18				
31	major capsid protein	1.00E-46	5.00E-49			2.00E-36	6.00E-38				
32	Hypothetical	4.00E-76	6.00E-28	7.00E-30	1.00E-33	8.00E-45	2.00E-37				
33	Hypothetical	6.00E-105	2.00E-54	4.00E-45	2.00E-38	8.00E-29	9.00E-23				
34	Hypothetical		3.00E-12								

35	Hypothetical		2.00E-31	4.00E-12		2.00E-24	5.00E-34				
36	N4-gp56 family major capsid protein	0	0	2.00E-155	0	3.00E-179	0				
37	secretion activator protein; lysozyme-like protein		7.00E-61								
38	Holin of 3TMs, for gene-transfer release		8.00E-18								
39	Hypothetical	7.00E-26									
41	Integrase	3.00E-179									
42	Arc family DNA-binding protein		1.00E-22								
52	Hypothetical	5.00E-31									
55	Hypothetical	2.00E-89	7.00E-34								
59	morphogenetic protein	8.00E-29	1.00E-27								
60	Hypothetical	2.00E-32									
64	C4-dicarboxylate ABC transporter substrate-binding protein	2.00E-165									
65	Transcriptional regulator	7.00E-39									
67	class I SAM-dependent methyltransferase	7.00E-91									
69	Hypothetical	1.00E-21									
72	XRE family transcriptional regulator		8.00E-14								
	TOTAL	30	27	14	13	16	16	1	0	0	1

Supplementary Table S6: Atu_ph08 gene products present in other *Agrobacterium* phages.

Atu_ph08 gene product	Atu_ph08 protein function	Phage Match	Identity (%)	E-value	Query cover (%)	Protein name	Phage gp #
gp4	Hypothetical	7-7-1	81	9e-40	59	Hypothetical protein	15
gp21	Helicase	Atu_ph03	36	4e-48	6	Cell wall hydrolyse	35
		Atu_ph02	36	4e-48	6	Cell wall hydrolyse	32
		Atu_ph07	31	3e-18	3	Mega protein	42
gp37	protein of unknown function DUF847	7-7-1	28	6e-28	91	Putative membrane protein	10
gp56	Predicted hydrolases of HD superfamily	Atu_ph04	29	3e-20	92	putative HD superfamily hydrolase	116
gp65	Transcriptional regulator	7-7-1	47	2e-17	47	Putative transcriptional regulator	68
gp67	Hypothetical protein	Atu_ph07	33	1e-20	80	Hypothetical protein	599
